# Water Environmental Capacity Calculation and Allocation of the Taihu Lake Basin in Jiangsu Province Based on Control Unit

**DOI:** 10.3390/ijerph16193774

**Published:** 2019-10-08

**Authors:** Juan Huang, Yong Pang, Xiaoqiang Zhang, Yifan Tong

**Affiliations:** 1 College of Hydrology and Water Resources, Hohai University, Nanjing 210098, Jiangsu, China; 2 Jiangsu Provincial Academy of Environmental Science, Nanjing 210036, Jiangsu, China; dr.xqzhang@gmail.com (X.Z.); tong57@purdue.edu (Y.T.); 3 Jiangsu Province Key Laboratory of Environmental Engineering, Nanjing 210036, Jiangsu, China; 4 College of Environment, Hohai University, Nanjing 210098, Jiangsu, China; ypang@hhu.edu.cn

**Keywords:** control unit, water environmental capacity, capacity allocation, Taihu Lake Basin in Jiangsu Province

## Abstract

The water quality target management of the control unit is a convenient and direct technology for water environment management and the development direction of water environment management in China, involving control unit division and water environment capacity calculation. Taking the Taihu Lake Basin in Jiangsu Province as an example, we propose herein the basic principle of the division of a regional control unit in a plain river network and the method of analyzing the rationality of the control unit division. On this basis, the Taihu Lake Basin in Jiangsu Province was divided into 70 control units. To calculate the water environmental capacity in the plain river network area, we established a water environmental capacity calculation framework based on multiple targets of lakes and rivers, and proposed the goal of water quality “double compliance” of the water environmental functional zone and the assessment section. For this study, we calculated the regional water environmental capacity using the mathematical model of the Taihu Lake Basin’s water environmental capacity, and the water environmental capacities of the 70 control units were allocated by the weight coefficient method, which established water area and functional division length. The research results described herein were applied to the pollution permit management of the Taihu Lake Basin in Jiangsu Province. It provides important technical support for the establishment of a pollution permit system based on the total capacity to improve environmental quality.

## 1. Introduction

Since the 1970s, extensive research on water environmental capacity, water functional division, a water quality model, a comprehensive water pollution prevention and control plan, and a sewage permit management system has been conducted in China. The combination of total emission control technology and water pollution control plans has gradually developed a water quality management technical system, focusing on the targeted total pollutant control technology, supplemented by total emission capacity control and industry total emission control [1,2]. Since the 10th Five-Year Plan, China has adopted a targeted total emission control system. According to the current pollution situation, the total emission control target is determined to a certain proportion. It has strong operability in management and is easy to implement. It has a positive effect on pollution control in China at a certain stage of development [3]. However, since the response relationship between the amount of pollutant discharge and the water quality of the receiving water is not considered, the pollutant reduction is inconsistent with the improvement of water quality, and the water quality improvement effect is not quite obvious [3].

Many advanced total emission control models have been proposed in developed countries, such as Japan’s basin total emission control plan, total maximum daily load (TMDL) in the United States, and the European Union (EU)’s water framework directive [4,5,6,7,8,9]. These management modes have detailed and specific provisions for the technical diagnosis of the sewage body, the determination of water quality indicators, the formulation, implementation, and evaluation of pollution control measures in the total control process, and the manner of ensuring the efficiency of water environment management [4].

The water quality target management of the control unit is a water quality management method based on the water environment management concept of “zoning, classification, grading, and staging”. It is in the water environment management hierarchy of “watershed - area - control unit - polluting source”, developed based on watershed total emission control and the control unit, and oriented to pollution sources [10,11,12,13,14,15]. The water quality target management of the control unit considers the characteristics of administrative divisions and watersheds, and overcomes the limitations of water quality management in a single area, thus providing specific and operational recommendations for pollution control. It can also refine water environment management targets by level as needed. It is conducive to investigating the achievement of water quality targets and the treatment effect of pollution sources. It is a convenient and direct water environment management technology, which is currently directing China’s water environment management development [16].

The water quality target management of the control unit includes two key technologies—control unit division and control unit water environmental capacity accounting [17]. Water environmental capacity refers to the capacity of a water body to contain pollutants under a certain water quality target, based on the water function area, and is the maximum allowable pollution load that meets the water quality standard [18]. It reflects the characteristics of the water environment to contain pollutants and the self-purification capacity of the water body [19]. In the context of total pollutant control implementation, research on the control of water environmental capacity and total pollutant discharge has become a hot topic [20,21]. At present, the calculation methods for water environmental capacity mainly include an analytical method, a model trial and error method, a system analysis method, an unascertained mathematical method, a probability dilution model method, and a matrix analysis method, etc. [22]. Liebman and Lynn [23], Ecker [24], and Loucks et al. [25] took parameters such as flow rate as deterministic variables in the study of water environmental capacity. Thomann and Sobel [26] and Revelle et al. [27] linearized an objective function and used an optimization model to calculate the emission and reduction of pollutants. Lohani and Thanh [28], Fujiwara et al. [29], and Eheart and Park [30] used a probabilistic constraint model to study the distribution of pollution load on the premise that it could cause excessive performance. Burn and McBean, Burn and Lence, and Cardwell and Ellis [31,32,33] considered the mutual constraints of multiple uncertainty factors, including pollution load, hydrology, and meteorology, and carried out pollution load calculation and distribution. Li and Morioka [34] calculated the allowable emissions using a mathematical model on the premise of taking into account the river’s transverse mixing non-uniformity. Therefore, the control unit is the basic implementation unit in the management of water quality targets in the basin. Based on this, water environmental capacity accounting can be carried out, which is of great significance for the scientific development of the total capacity allocation plan of the control unit and the management of the discharge permit.

The Taihu Lake Basin in Jiangsu Province is one of the most developed regions in China. During the rapid economic development, the water ecosystem was damaged to varying degrees due to the impact of human activities, and water pollution is very serious. In response to the water pollution problem in Taihu Lake and its watershed, researchers have conducted a number of fruitful studies [35,36,37,38,39], and achieved innovative results. The manager also took a number of measures for pollution control and governance, but the problem of water pollution has not yet been solved fundamentally. A basin water quality target management system must be established based on the water source’s pollution control unit to achieve the management of “zoning, classification, grading, and staging” [40]. Currently, there are fewer water system capacity calculations at the river basin level. For this study, we focused on the complexity of the river network in the Taihu Lake Basin, the indefinite water flow, the unclear watershed boundary, the frequent regulation of water conservancy projects, and the staggered superposition of pollution sources, and propose herein the basic principle of the control unit division of a plain river network and the rationality of the control unit division. On this basis, the Taihu Lake Basin in Jiangsu Province was divided into 70 control units. To calculate the water environment capacity in the plain river network area, we established a water environmental capacity calculation framework based on multiple targets of lakes and rivers, and proposed the goal of water quality "double compliance" of the water environmental functional zone and the assessment section, and then calculated the regional water environmental capacity using the mathematical model of the Taihu Lake Basin’s water environmental capacity. We also allocated the water environmental capacities of the 70 control units by the weight coefficient method, which established water area and functional division length. The research results described herein were applied to the pollution permit management of the Taihu Lake Basin in Jiangsu Province. It provides important technical support for the establishment of a pollution permit system based on the total capacity to improve environmental quality.

## 2. Study Area

**Figure 1:**
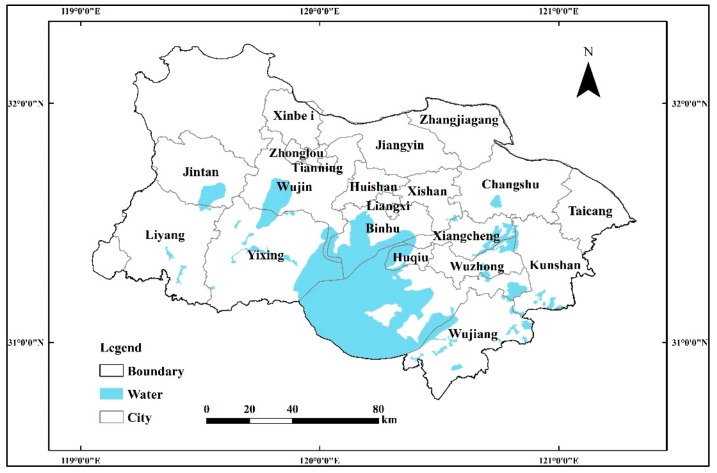
Study area and control section distribution.

The Taihu Lake Basin in Jiangsu Province is located in the southern part of the province, adjacent to Zhejiang Province in the south and Shanghai City in the east. The basin is situated between 30°48′36″ and 32°13′2″ N and 118°42′ and 121°18′ E. Covering an area of 19,399 km^2^, this basin crosses over 21 administration districts, as depicted in Figure 1.

The area is a typical plain river network area. The river network is interlaced and there are many lakes. The total length of the rivers is approximately 120,000 km and the river network density is 3.3 km/km^2^. The actual river flows of the total research area are 17.6 billion m^3^, and the Yangtze River and Taihu Lake are the main sources of water for the basin. The average annual rainfall is 1,181 mm, of which 60% is concentrated during the months of May–September.

The water quality of the study area is poor. Based on the water quality data, including chemical oxygen demand (COD), ammonia nitrogen (NH_3_-N), and total phosphorous (TP), from 65 conventional water quality monitoring stations from 2011 to 2012 provided by the Environmental Monitoring Centre of Jiangsu Province, the water quality categories of the study area are mainly Grades III to V. According to the “Environmental Quality Standards for Surface Water (GB3838-2002)”, all the surface water can be classified into five water quality grades deteriorating from Grade I to Grade V. In March 2003, the people’s government of Jiangsu Province approved the “Classification Scheme of Surface Water Environment Functional Area in Jiangsu Province”, wherein all the important cross-sections in Jiangsu Province were required to achieve the relevant standard. Based on the water monitoring data of all the important cross-sections from 2011 to 2012, the water quality is below the legal standard in 30% of the samples, as presented in Table 1. The types of pollution sources in this area include industry, residential pollution, farmland, and livestock. According to the statistics on pollutant discharge provided by the Environmental Protection Bureau, there were 2,483 industrial enterprises in the area, and the amount of wastewater discharge in the study area was 65.271 million t in 2011.

## 3. Method

### 3.1. Outline

The main research methods of this study are as follows. (i) Division of the control unit, where the control unit is regarded as the basic management unit. (ii) Calculation of the water environment capacity in the plain river network area. A water environmental capacity calculation framework was established based on multiple targets of lakes and rivers, and “double compliance” was proposed. The term “double compliance” means the water quality target of the water environment function area reaches the standard and the water quality target of the assessment section is up to the standard. (iii) Control of the unit water environmental capacity allocation, where the weight coefficient method was used. According to the water area and the length of the functional area, the regional water environment capacity is distributed to each control unit.

### 3.2. Division of Control Unit

#### 3.2.1. Control Unit Division

The control unit is the area where the main pollution has the most prominent influence on the water quality of the control section [41,42,43]. During the 11th Five-Year Plan period, Gao and Gao divided the Jiangsu area of the Taihu Basin into 38 control units [44,45]. Given that the locations of the assessment sections in Jiangsu Province were not clear during the 11th Five-Year Plan period, the division of the control unit had little relation with the assessment sections. As a result, there was no corresponding control section for the supervision and management of the control unit division. Beginning in 2012, the assessment sections were built and operated. Therefore, the division results of the control unit during the 11th Five-Year Plan period did not correspond to the assessment sections.

The control section consisted of 65 sections and 26 drinking-water sources. During the 12th Five-Year Plan period, 65 automatic monitoring stations were set up to be used as the assessment sections by the Environment Monitoring Center of Jiangsu Province. The improvement in the monitoring system offered strong support for water environment management and simultaneously provided an important referential basis for the modification of the control unit. The drinking-water sources were the origins of both the domestic water and the industrial water. Therefore, one of the important objectives of control unit management was to ensure high water quality in the drinking-water sources. The first-level protection zone in the Taihu Basin was directly related to the water environmental quality of Taihu Lake. The first-level protection zone means the range of the Taihu Lake body, the area five kilometers along the lakeshore, the rivers going into the lake 10 kilometers upstream, and the range of one kilometer on both sides of the river. Therefore, the water quality compliance of the intersection of the Taihu first-level protection zone and the important water system is also an important assessment factor for the management of the control unit. Considering the location of the control section, we developed a method based on the results of the control unit division during the 11th Five-Year Plan period. The basic principles of the control unit division methods adopted in this study are described as follows. (i) The first principle is to regard the location of the control section in the study area during the 12th Five-Year Plan as a reference. During the 12th Five-Year Plan’s implementation period, the Environmental Monitoring Center of Jiangsu Province established a total of 65 automatic monitoring stations as assessment sections. Additionally, there were 26 drinking-water sources and 20 sections of the boundary between the first reserve and the rivers in the study area. On the premise of having clarified the location of the assessment sections, drinking-water sources, and sections of the boundary between the first reserve and the rivers, this study made adjustments and improvements to the existing division results of the control unit in the study area. Moreover, it was essential to ensure that each control unit had at least one control section. (ii) The second principle is to consider water conditions. There are many rivers and lakes in the study area. Owing to the influence of the tides, most rivers at the downstream boundary are bi-directional. Therefore, the impacts of water quality in the control sections can be varying and complicated. Furthermore, the main hydrological conditions of flow direction and velocity need to be considered in the division of the control unit. (iii) The third principle is to consider the country or district administration boundary. To enable easy administration management, the regulated control unit boundary must primarily consider the administration boundary. As for the administration boundaries devoid of regulations, further improvements must be made in accordance with the river system distribution.

#### 3.2.2. Rationality Analysis

By calculating the influential weight of the control unit internal pollution load against the control section water quality, the rationality of the control unit division can be determined, which is expressed as follows [46]:

(1) $$\alpha_{i}=\frac{C_{i}/W_{i}}{C_{i}/W_{i}+C_{0}/W_{0}}$$

where *α_i_* is the influential weight of the control unit internal pollution load against the control section water quality (%), *W_i_* is the internal pollution load of the control unit, *C_i_* is the influential water concentration value of the control unit internal pollution source against the control section water quality, *W_0_* is the external pollution load of the control unit, and *C_0_* is the influential water concentration value of the control unit external pollution source against the control section water quality. 

#### 3.2.3. Calculation of Water Environmental Capacity in Plain River Network Area

1.  River network water environment capacity based on the multiple targets of lakes and rivers

**Figure 2:**
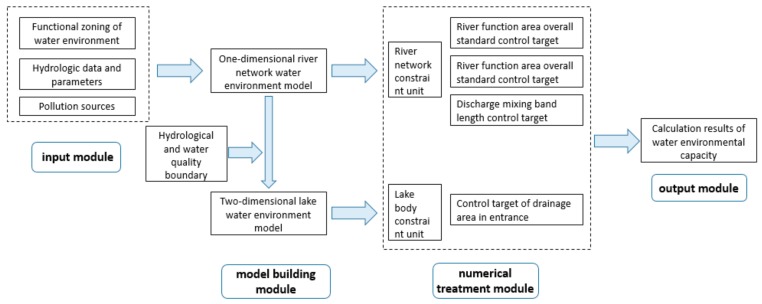
Water environment capacity calculation system based on multiple targets of rivers and lakes.

The research area is a complex river and lake system. There are two types of water bodies, namely rivers and lakes. Therefore, it is necessary to consider the dual targets of rivers and lakes when calculating the water environment capacity of the river network area. The impact of the river network input to the lake water environment must be considered while ensuring the water quality targets of the river network. The impact of sewage in the river network area on the Taihu Lake body must be incorporated into the constraint system of the water environment capacity calculation in the river network area. This study proposes a calculation system for the water environment capacity in the river network area based on multiple targets, such as river function compliance in the river network, confinement of sewage outlet, control section compliance, and pollution control of the lake inlet. This system consists of four modules, namely input, numerical simulation, data processing, and result output, as illustrated in Figure 2.

The input and output modules, respectively, obtain the initial input and final result output of the river and lake system water environment target, hydrological condition, and pollution source intensity. The numerical simulation module mainly obtains the numerical simulation of the water flow and water quality process of the river and lake system through the mathematical model of the coupled water environment of the river network. The data processing module is essential in the computing system, and mainly includes the river network constraint unit and the lake body constraint unit.

The river network constraint unit considers the following three factors as the calculation constraint conditions—the overall water quality of the river network area, the water quality of the control assessment section, and the length of the pollution discharge zone of the sewage outlet. The lake body constraint unit considers the influence of the material input of the river network area on the lake pollution mixed zone as the calculation constraint condition.

2.  River network constraint unit control target calculation method

The river network restraint unit control target refers to the water environment capacity under conditions of “double compliance” for the overall water quality compliance control and control section water quality compliance control. The calculation method for the overall water quality compliance of the functional area is based on the zero-dimensional water quality model, and the calculation result is independent of the location of the pollution source. Calculations are carried out as follows:

(2) $$E_{ij}=Q_{0ij}\left(A_{sij}-A_{0ij}\right)+KV_{ij}A_{sij},$$

(3) $$E=\sum_{j=1}^{n}\sum_{i=1}^{m}\beta_{ij}\times E_{ij},$$

where *E_ij_* is the water environment capacity in the calculation. The minimum space calculation unit in the calculation is the river section (the river section is the river channel between the two nodes), and the minimum time calculation unit is a day; *Q_0ij_* and *V_ij_* are designed hydrological conditions, calculated by the Taihu River network model [47,48]; *A_si_*_j_ is the functional water quality target; *A_0ij_* is the upstream water quality concentration; *K* is the water quality degradation coefficient; and *β_ij_* is the uneven mixing coefficient. The value refers to the range of uneven mixing coefficient of general rivers [49,50]. The water environmental capacity value of the minimum spatial extent and minimum length of time of each water body is calculated according to Equation (2). Then, the water environmental capacity value of the study area is calculated according to Equation (3).

The calculation method for the water quality compliance of the control section is based on one-dimensional and two-dimensional water quality models, and the calculation results are related to the pollution source. To calculate the water environmental capacity of the control section, the water quality response relationship between the sewage outlet of each control unit and the control section must first be established, and then the water environmental capacity under the conditions where the control section meets the standard is calculated. The one-dimensional steady-state model is used to calculate the water quality standard of the regional control section. The basic model is given as follows:

(4) $$M=\frac{P+Q_{0}D_{0}}{Q_{0}}exp(-k\frac{x}{86400u}),$$

where *M* is the concentration of water pollutants at distance *x*; *k* is the water quality degradation coefficient; *u* is velocity; *x* is the longitudinal distance between the pollution source and the calculated section; *P* is the emission of pollutants; *D_0_* is the boundary water concentration; and *Q_0_* is the river flowrate, *Q_0_ = B_hu_*, where *B* is the river width and *h* is the average depth of the riverway.

3.  Lake body constraint unit control target calculation method

As the pollutants received by Taihu Lake are primarily brought into the lake by rivers, this paper considers the entrance to the lake channel as a sewage outlet. The amount of permitted pollutant discharge under the control target of the Taihu Lake Zhushan Bay is calculated by controlling the area of a single pollution zone. The mathematical model of two-dimensional unsteady water quantity and water quality is used to calculate the downward pollution zone of different wind speeds. Then, the permitted volume of pollution discharge of the pollution zone, that is, the target value of constraint unit of the Zhushan Bay river network area, is set. The calculation equation for the control target value of the Shushan Bay Lake body restraint unit is as follows:

(5) $$F=\sum_{j=1}^{b}\left(\gamma_{j}\sum_{i=1}^{n}N_{ij}\right)+\Delta N,$$

where *F* is the amount of pollutants allowed to be discharged (t/a); *N_i_*_j_ is the allowable discharge of a single sewage outlet in a certain wind direction (t/a), and is controlled by the area of the pollution zone (1–3 km^2^); *γ_j_* is each wind direction wind speed frequency (%); *n* is the number of sewage outlets (a); *b* is the number of different wind speeds in different wind directions (a); and Δ*N* is the allowable emission correction value (t/a).

#### 3.2.4. Analysis of Rationality of Regional Water Environment Capacity Calculation

The regional pollutant reduction rate is compared with the water quality that exceeds the standard, and the rationality of the calculation result of the water environment capacity is analyzed. The calculation equation for the pollutant reduction rate is as follows:

(6) $$\mathrm{PR}=\frac{\mathrm{PE}-EC}{\mathrm{PE}}\times 100\%,$$

where *PR* is the pollutant reduction rate (%); *PE* is amount of pollutants entering the river in 2011; *EC* is the water environmental capacity which means the maximum allowable amount of pollutants.

#### 3.2.5. Control Unit Water Environment Capacity Allocation Method

The calculation equation for the water environment capacity allocation of each control unit is given by the following:

(7) $$\lambda_{j}=\frac{\sum_{i=1}^{5}(S_{j}\times \frac{l_{ij}}{\sum_{i=1}^{5}l_{ij}}\times R_{si})}{\sum_{i=1}^{5}(S\times \frac{l_{i}}{\sum_{i=1}^{5}l_{i}}\times R_{si})}\;,$$

(8) $$Y_{j}=\lambda_{j}\times Y_{t},$$

where *λ_j_* is the environmental capacity weight occupied by a control unit j; *Y_t_* is the total water environment capacity of the study area; *Y_j_* is the water environmental capacity of a control unit j; *R_si_* is the i-type water quality standard in terms of concentration; *S_j_* is the water area of a control unit j; *l_ij_* is the total length of the *i*-type water function area of a control unit j; *S* is the total water area of the study area; and *l_i_* is the total length of the i-type water function area of the study area, namely:

(9) $$S=\sum_{j=1}^{n}S_{j},$$

(10) $$l_{i}=\sum_{j=1}^{n}l_{ij}.$$

The water area of each control unit was obtained by cutting and dividing the control unit and the GIS remote sensing map. The length of each control unit function area was obtained by the control unit and the functional area MapInfo statistics. The functional lake areas were uniformly converted into perimeter statistics.

## 4. Results and Discussion

### 4.1. Control Unit Division Results

**Figure 3:**
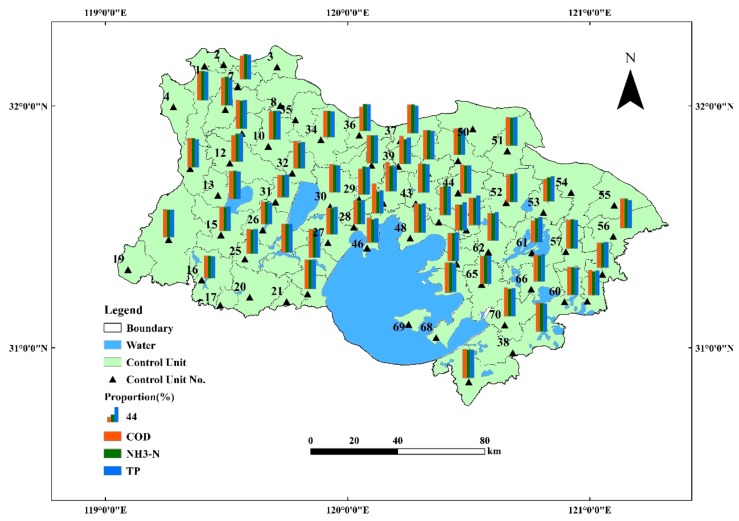
Control unit division results and weight of control unit internal pollution against control section water quality.

Based on the divided results of the control units in the Taihu Basin during the 11th Five-Year Plan, this paper adopted the division method to further improve control unit division in the study area, which considers the country and district administration boundaries, automatic monitoring system, drinking-water source, sections of the boundary between the first reserve and the rivers, and basin and water conditions. After combination, refinement, and modification of the boundary, this paper divided the study area into 70 control units. The results of control unit division are shown in Figure 3.

Based on the premise of clarifying the location of assessment sections, drinking-water sources, and sections of the boundary between the first reserve and the rivers, this paper made improvements to the existing division results of the control unit in the study area. Moreover, it is significant to make sure that each control unit has one control section. Compared with the control unit division results during the 11th Five-Year Plan, the results in this paper are more convenient for environmental management.

### 4.2. Rationality Analysis of Control Unit Division Results

The control unit is the area in which pollution prominently influences the water quality of the control section. By calculating the influential weight of the control unit internal pollution load against control section water quality, the rationality of the control unit division is determined. According to the pollution load materials, the water condition materials, and control section information in the study area, the influential weight of control unit internal pollution against control section water quality can be calculated. The calculation results are shown in Figure 3. Based on the maximizing integrity of the water system, the scale of the divided control unit is used to reflect the pollution status in the area. According to the calculation results, the influential weight of each control unit internal pollution source in the control section of water quality ranges between 65% and 88%. This implies that the internal pollution source in the control unit is the main influencing factor of control section water quality. The control section water quality can be ensured by controlling the internal pollution source in the control, so the division of each control unit remains rational. Among others, the internal rivers of the sixteen control units, with codes 3, 8, 35, 50, 54, 55, 68, and 69, all belong to river sources, and those with codes 1, 2, 4, 14, 17, 19, 20, and 21 all belong to hills. No pollution load exists outside the control unit.

### 4.3. Calculation Results of Regional Water Environment Capacity

Under the conditions of 90% hydrological guarantee rate in the Taihu Basin, and the 2020 water quality target in the surface water environment functional division of Jiangsu Province, and according to the calculation method of water environment capacity, the environmental capacity of chemical oxygen demand (COD), ammonia nitrogen (NH_3_-N), and total phosphorus (TP) in the Taihu Lake Basin of Jiangsu Province was 237,500 t/a, 21,000 t/a, and 0.4 million t/a, respectively. The calculation results are shown in Table 2.

### 4.4. Rationality Analysis of the Results of Regional Water Environmental Capacity Calculation 

The volume of pollutants entering the river in 2011 in the Taihu Lake Basin of Jiangsu Province, the calculated water environment capacity, the reduction rate of pollutants entering the river, and the 2011 water quality exceeding rate measured by the water conservancy department are summarized in Table 2.

**Figure 4:**
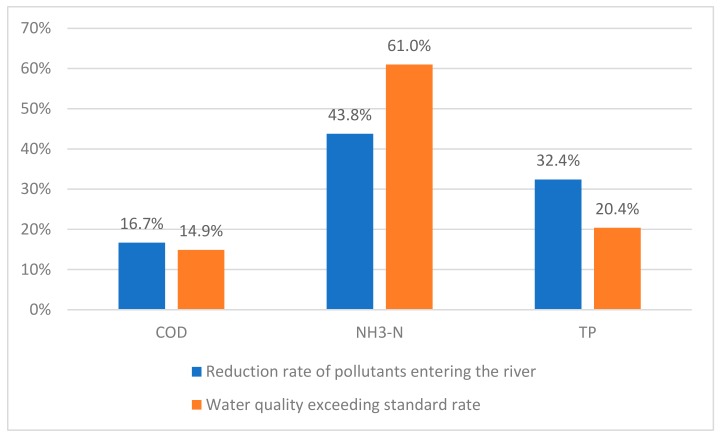
Comparison of calculated inflow rate reduction rate and water quality over-standard rate.

The calculated 2011 pollutant reduction rate and the 2011 water quality exceeding standard rate provided by the Jiangsu Hydrology and Water Resources Survey Bureau are shown in Figure 4. The difference between the inflow quantity reduction rate of calculated COD, ammonia nitrogen, and total phosphorus and water quality over-standard rate are basically within 20%, and the reduction rate is consistent with the water quality over-standard rate, indicating that the calculated value of water environment capacity is reasonable.

### 4.5. Control Unit Water Environment Capacity Calculation Results

The water area of each control unit was obtained by cutting and dividing the control unit and GIS remote sensing map. The length of each functional unit’s functional area was obtained from the control unit and the functional zoning MapInfo statistics. The functional areas of the lake are uniformly converted into perimeter statistics. According to the calculation results of the water environment capacity of the area, the water area of each control unit, and the length of the functional area, the water environment capacity of each control unit is calculated using Equations (6) and (7). The calculation results are shown in Table 3.

### 4.6. Application of Sewage Disposal License Management System

During construction of the dynamic management system for the permitted sewage limit in the Taihu Basin of Jiangsu Province, the calculation results of the control unit division and its environmental capacity were fully utilized. In the sewage permit management system, users can query the location and scope, the in-unit distribution of the sewage outlets, the location of the water quality section, the environmental capacity information, the information of the issued pollutant discharge permit, and the remaining environmental capacity information of each control unit. The environmental protection department can track the discharge permit status and environmental capacity of each control unit in real time. When the discharge amount of the pollutant discharge permit issued by a control unit is close to or has exceeded the environmental capacity of the area, the environmental protection department may adopt a freezing measure to suspend the issuance of the discharge permit in the control unit.

## 5. Conclusions

First, the division of the control unit regarded as the basic unit of water pollution control must fully take the river direction and water system integrity characteristics of water systems in the plain river network area into consideration. Based on control unit division methods, we divided the Taihu Lake Basin in Jiangsu Province into 70 control units. The influential weight of control unit internal pollution load against control section water quality ranges between 65% and 88%. Thus, the division results of the control units are basically rational.

For this study, we established a water environment capacity calculation system based on multiple targets of rivers and lakes, and proposed the water environment functional zone and the assessment section water quality “double compromise” target requirements. Based on the mathematical model of the Taihu Lake water environment, the typical hydrological design conditions and appropriate model parameters were selected to calculate the water environment capacity of the Taihu Lake Basin in Jiangsu Province. Under the conditions of 90% guaranteed hydrological rate and 2020 water environment functional zoning, the environmental capacity of COD, ammonia nitrogen, and total phosphorus water in the Taihu Lake Basin of Jiangsu Province was 237,500 t/a, 21,000 t/a and 4,000 t/a, respectively. We propose to allocate the regional water environmental capacity to 70 control units according to the weight coefficient method of water area and functional division length. Taking the control unit no. 20 as an example, the water area of the control unit is determined to be 36.2 km^2^ by GIS remote sensing map cutting. 

For this study, we established a water environment capacity calculation system based on multiple targets of rivers and lakes, and proposed the water environment functional zone and the assessment section water quality “double compromiseȁ target requirements. Based on the mathematical model of the Taihu Lake water environment, the typical hydrological design conditions and appropriate model parameters were selected to calculate the water environment capacity of the Taihu Lake Basin in Jiangsu Province. Under the conditions of 90% guaranteed hydrological rate and 2020 water environment functional zoning, the environmental capacity of COD, ammonia nitrogen, and total phosphorus water in the Taihu Lake Basin of Jiangsu Province was 237,500 t/a, 21,000 t/a and 4,000 t/a, respectively. We propose to allocate the regional water environmental capacity to 70 control units according to the weight coefficient method of water area and functional division length. Taking the control unit no. 20 as an example, the water area of the control unit is determined to be 36.2 km^2^ by GIS remote sensing map cutting. 

To develop the sewage management permit dynamic management system of the Taihu Lake Basin in Jiangsu Province, the calculation results of control unit division and control unit environmental capacity were used. The environmental protection department may suspend the issuance of the pollutant discharge permit of the control unit when the total discharge of the pollutant discharge permit issued by a control unit is about to exceed or has exceeded the environmental capacity of the control unit. This method provides important technical support for the establishment of a pollutant discharge permit system based on the total capacity to improve environmental quality.

## Figures and Tables

**Table 1:** Water quality information for the Taihu Lake Basin in Jiangsu Province (unit: %). COD, chemical oxygen demand; NH_3_-N, ammonia nitrogen; TP, total phosphorous.

Year	I	II	III	IV	V	Worse Than V	Single Factor Exceeding Rate			Exceeding Rate
							COD	NH_3_-N	TP	
2011	0.9	18.6	35.6	26.8	8.4	9.7	10.02	44.20	18.35	44.20
2012	2.4	25.5	40.0	17.8	7.1	7.2	9.54	30.16	27.03	30.16

Notes: 1. According to the “Environmental Quality Standards for Surface Water (GB3838-2002)”, all the surface water can be classified into five grades deteriorating from Grade I to Grade V. 2. ”%” is in all sections, the proportion reaching this grade.

**Table 2:** Rationality analysis of the calculation value of water environment capacity in cities in the Taihu Lake Basin in Jiangsu Province (unit: 10,000 t/a).

Cities	COD				NH_3_-N				TP			
	Inflow Quantity	Water Environmental Capacity	Inflow Quantity Reduction Rate	Water Quality Standard Exceeding Rate	Inflow Quantity	Water Environmental Capacity	Inflow Quantity Reduction Rate	Water Quality Standard Exceeding Rate	Inflow Quantity	Water Environmental Capacity	Inflow Quantity Reduction Rate	Water Quality Standard Exceeding Rate
Wuxi	6.3	5.4	14.0%	18.9%	0.6	0.5	14.0%	70.3%	0.14	0.11	24.8%	17.0%
Changzhou	7.1	6.0	15.7%	22.1%	0.9	0.4	54.9%	69.0%	0.17	0.08	52.3%	27.2%
Suzhou	15.1	12.3	18.3%	3.6%	2.2	1.1	47.8%	43.8%	0.31	0.23	24.9%	17.0%
Total	28.5	23.7	16.7%	14.9%	3.7	2.1	43.8%	61.0%	0.62	0.42	32.4%	20.4%

**Table 3:** Water environment capacity table of the Taihu Basin control units, Jiangsu Province (unit: t/a).

Control Unit No.	COD	NH_3_-N	TP	Control Unit No.	COD	NH_3_-N	TP
1	394	26	5	36	2510	214	43
2	250	15	3	37	2527	233	47
3	404	24	5	38	11,051	946	189
4	945	62	12	39	3881	361	72
5	1486	92	18	40	6456	596	119
6	3145	207	41	41	4377	407	81
7	418	28	6	42	2534	261	52
8	612	38	8	43	1825	188	38
9	1632	108	22	44	2340	241	48
10	2427	160	32	45	1169	120	24
11	934	57	11	46	517	47	9
12	6168	411	82	47	244	24	5
13	9536	636	127	48	1627	159	32
14	6047	443	89	49	155	16	3
15	6907	505	101	50	9400	865	173
16	2040	119	24	51	10,516	991	198
17	119	9	2	52	8478	783	157
18	5389	502	100	53	12,492	1181	236
19	341	21	4	54	5831	539	108
20	1321	133	27	55	6233	576	115
21	143	15	3	56	8192	737	147
22	1118	105	21	57	4538	398	80
23	2656	278	56	58	2220	197	39
24	3835	413	83	59	2896	271	54
25	3835	413	83	60	8396	785	157
26	6793	732	146	61	7019	648	130
27	4426	464	93	62	2209	244	49
28	3330	223	45	63	207	23	5
29	1595	107	21	64	600	66	13
30	8677	582	116	65	2413	267	53
31	1704	114	23	66	3504	388	78
32	2290	154	31	67	277	25	5
33	5878	394	79	68	1499	116	23
34	3782	245	49	69	197	15	3
35	833	55	11	70	9776	933	187
